# Association between the Hepatic Lipase Promoter Region Polymorphism (-514 C/T) and the Presence and Severity of Premature Coronary Artery Disease

**Published:** 2017-07

**Authors:** Hamidreza Goodarzynejad, Mohammadali Boroumand, Mehrdad Behmanesh, Shayan Ziaee, Arash Jalali, Leyla Pourgholi

**Affiliations:** 1 *Tehran Heart Center, Tehran University of Medical Sciences, Tehran, Iran.*; 2 *School of Biological Sciences, Tarbiat Modares University, Tehran, Iran.*

**Keywords:** *Coronary artery disease*, *Young adult*, *Polymorphism, genetic*

## Abstract

**Background: **Hepatic lipase (HL) plays a crucial role in lipid metabolism, but there is debate about whether HL acts in a more pro- or more anti-atherogenic fashion. We aimed to examine the relationship between the -514 C/T polymorphism within the HL gene (LIPC) and the risk of angiographically determined premature coronary artery disease (CAD).

**Methods: **Four hundred seventy-one patients with newly diagnosed angiographically documented (≥ 50% luminal stenosis of any coronary vessel) premature CAD were compared to 503 controls (subjects with no luminal stenosis in coronary arteries). A real-time polymerase chain reaction and high-resolution melting analysis was used to distinguish between the genotypes.

**Results: **There was no significant difference in the distribution of -514 C/T genotypes between the 2 groups in the whole population or in the men, but the examined polymorphism was found to be associated with the presence of CAD in the women (p value = 0.029). After the application of a multiple logistic regression model, the minor T allele of the LIPC gene was not found to be independently associated with the presence of CAD either in the total population (adjusted OR = 0.97, 95% CI = 0.75-1.25; p value = 0.807) or in the women (adjusted OR = 0.91, 95% CI = 0.59-1.40; p value = 0.650) and in the men (adjusted OR = 1.15, 95% CI = 0.81-1.64; p value = 0.437) separately.

**Conclusion: **Our findings suggest that there is no relationship between the LIPC -514 C/T and the risk of premature CAD or its severity in patients undergoing coronary angiography.

## Introduction

Hepatic lipase (HL) plays an essential role in lipid metabolism by hydrolyzing triglycerides and phospholipids in circulating lipoproteins and by acting as a ligand/bridging factor for receptor-mediated lipoprotein uptake. Despite its crucial function, there is controversy as to whether HL acts in a more pro- or more anti-atherogenic manner. Arguments for an anti-atherogenic role of HL include one contending that human HL expression reduces the deposition of aortic cholesterol by 42% in cholesterol-fed transgenic mice.^1^ In addition, patients with familial deficiency of HL develop premature coronary artery disease (CAD).^2^ Arguments for a pro-atherogenic role include the negative association between HL activity and the plasma levels of anti-atherogenic high-density lipoprotein cholesterol (HDL-C)^3^ and the positive association between HL activity and the plasma levels of small, dense low-density lipoprotein particles in those with CAD.^4^ Furthermore, mice deficient in apolipoprotein E develop less atherosclerosis provided that they are in addition made HL deficient.^5^ The association between HL activity and CAD does not necessarily imply a contribution of HL to CAD risk, whereas an association between a genetically induced variant of HL expression and CAD is more likely to be indicative of a causality relationship.

The HL gene (LIPC) is located on chromosome 15 (q21-q23). HL deficiency, caused by rare LIPC mutations, is generally linked to increased CAD risk.^6^ However, due to the small number of the affected individuals, its contribution to CAD is not significant. For the general population, common functional variants in the LIPC gene are of interest. It has been demonstrated that the single-nucleotide polymorphisms (SNPs) in the LIPC gene are related to plasma lipid levels, cardiovascular risk factors, and elevated risks of CAD.^7^^-^^10^ The most frequently studied SNP, which is in complete linkage disequilibrium with several other SNPs in this region, is described as –514C > T or –480C > T.^11^^, ^^12^ This SNP is located in the promoter region and has been suggested to be associated with a lower HL activity, 12 which is shown to be a risk factor for CAD.^13^ Nonetheless, the effects of this SNP on the presence of CAD are controversial.^14^ The association between the –514C >T promoter variant and the occurrence of CAD has been reported in a small population of Iranian patients with general CAD.^15^ The aim of this study was to investigate this association among a relatively large population of Iranian patients with premature CAD, defined as the presence of coronary artery atherosclerotic lesions in men aged below 45 years and women aged below 55 years.^16^

## Methods

This case–control study was carried out on 560 patients with newly diagnosed angiographically documented premature CAD and the same number of controls with normal coronary arteries matched for gender and age. Cases were consecutively enrolled, between May 2009 and July 2011, from those with CAD documented by coronary angiography. A sex-matched sample of 560 unrelated controls (274 men, 45.7%) was randomly selected from a pool of 1.547 young patients (419 men and 1.128 women) who, between June 2004 and July 2011, were consecutively admitted to our hospital for elective coronary angiography. The exclusion criteria were previous history of acute myocardial infarction, stent implantation, cardiopulmonary resuscitation, and coronary artery bypass graft surgery. In addition, patients taking lipid-lowering drugs and those with minimal CAD (coronary lesions with < 50% luminal stenosis) were excluded. Final analyzed patients were age-matched with controls at group level (Table 1). In agreement with the Declaration of Helsinki for research involving human subjects, all the participants provided written informed consent explicitly granting permission for relevant clinical data gathering and DNA analysis. The local ethics committee approved the study protocol. Coronary angiographies were done via the percutaneous femoral approach using standard techniques. All the cases had at least 50% luminal stenoses in at least 1 coronary artery or major branch segment in their epicardial coronary tree. The controls had no luminal stenosis on coronary angiography. The definitions for the analyzed risk factors of CAD including dyslipidemia, hypertension, cigarette smoking, family history of CAD, diabetes, and the body mass index (BMI) have been reported elsewhere.^17^^, ^^18^

**Table 1:** Baseline characteristics in the study population based on coronary artery disease presence*

	non-CAD (n=503)	CAD (n=471)	P value
Age (y)	45.51±6.15	45.47±5.91	0.927
BMI (Kg/m^2^)	29.65±4.77	29.65±5.31	0.998
Male sex	225 (44.7)	224 (47.6)	0.376
Diabetes mellitus	67 (13.3)	154 (32.7)	< 0.001
Hypertension	173 (34.4)	255 (54.1)	< 0.001
Smoking status			< 0.001
Current smoker	85 (16.9)	121 (25.7)	
Ex-smoker	43 (8.5)	49 (10.4)	
Non-smoker	375 (74.6)	301 (63.9)	
Family history of CAD	140 (27.8)	198 (42.0)	< 0.001
Dyslipidemia	286 (56.9)	342 (72.6)	< 0.001
Total cholesterol (mg/dl)	185.87±42.01	186.35±54.12	0.879
HDL-cholesterol (mg/dl)	44.07±11.45	40.29±12.00	< 0.001
LDL-cholesterol (mg/dl)	115.76±35.65	116.74±43.88	0.712
Triglyceride (mg/dl)	172.43±110.60	192.02±116.74	< 0.001
Fasting blood sugar (mg/dl)	106.49±36.59	128.82±61.54	< 0.001
Creatinine (mg/dl)	0.89±0.61	0.86±0.35	0.478
LVEF (%)	55.57±7.96	51.80±9.43	< 0.001

BMI, Body mass index; CAD, Coronary artery disease; HDL, High-density lipoprotein; LDL, Low-density lipoprotein; LVEF, Left ventricular ejection fraction

* Data are presented as mean±SD or n (%).

Between June 2004 and July 2011, for the purpose of creating a DNA-bank of patients with premature CAD and controls, all young patients (age ≤ 45 y for men and age ≤ 55 y for women at disease onset) admitted to our center for elective coronary angiography were asked to provide a sample of whole blood for DNA extraction. The blood samples were placed in tubes containing ethylenediaminetetraacetic acid (EDTA) and were stored deep-frozen until later use. Genomic DNA was extracted from leukocytes using the buffy coat of these samples. DNA extraction was carried out using the standard “salting out” method. DNA quantity was evaluated by calculating absorbance at λ = 260 nm and DNA quality by a ratio of λ = 260/280 nm being close to 1.8. The purified DNA was stored in Tris-EDTA buffer (pH = 8.0) at -70 °C until further analysis.

**Figure 1:**
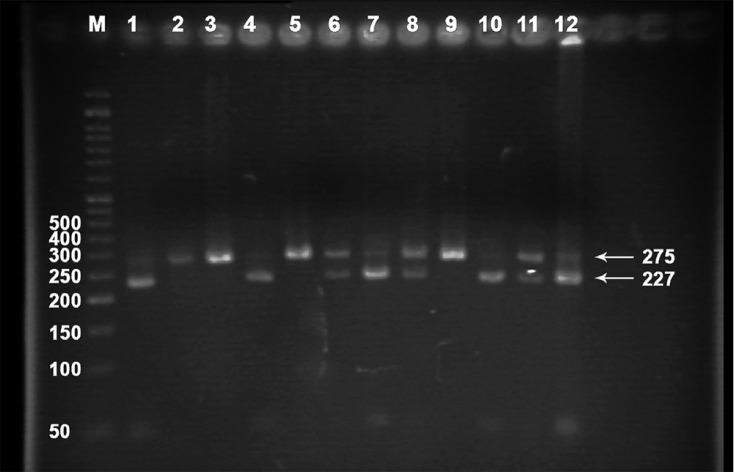
Agarose gel electrophoresis for the rs1800588 polymorphism of the hepatic lipase (LIPC) gene (C > T). The 275-bp polymerase chain reaction (PCR) product was digested by the Hin1II (NlaIII) restriction enzyme. The TT variant produced 2 fragments with 227 bp and 48 bp, while the heterozygote CT produced 3 fragments of 275 bp, 227 bp, and 48 bp. The CC variant produced 1 fragment of 275 bp. The 48-bp fragment was not visible in the gel due to its fast migration speed.

**Figure 2:**
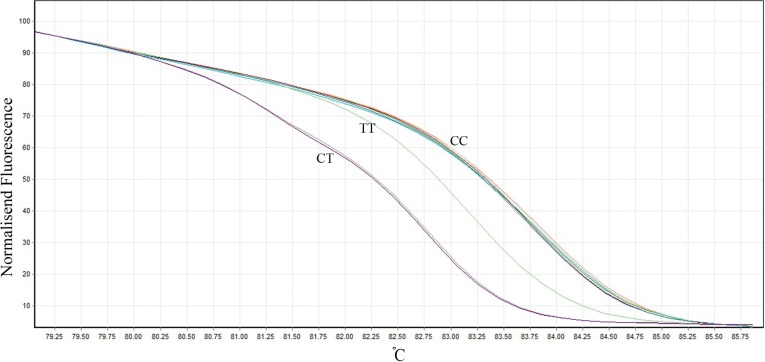
Graph of normalized fluorescence by temperature

Real-time polymerase chain reaction (PCR) and high-resolution melting (HRM) analysis was applied for the genotype analysis using a Corbett Rotor-Gene 6000 real-time rotary analyzer (Corbett Life Science Pty. Ltd., Mortlake, NSW, Australia). One set of primers based on common HRM specifications was designed using Beacon Designer software, version 7.0 (Premier Biosoft International, Palo Alto, CA, USA), and it was synthesized by Bioneer Inc. (Daejeon, Korea). The final PCR product length was 120 bp using the following primers: forward primer was 5′- TGTTTACTCTAGGATCACCTCTCAATG -3′ and the reverse was 5′- TGATGCTTGTGGTCAAAGTGTG -3′. The final reaction mixture contained Type-it HRM Master Mix (QIAGEN NV, Venlo, Netherlands) with intercalating DNA-binding Evagreen dye (Biotium, Inc. Hayward, CA, USA), DNA polymerase (5U/mL), primer mix (0.4 μM of each primer), genomic DNA (50 ng), and RNase Free Water (QIAGEN NV) in a total volume of 20 μL. The PCR cycling conditions consisted of an initial denaturation at 95 °C for 15 minutes, followed by 37 cycles of denaturation (96 °C for 20 sec), primer annealing (62 °C for 20 sec), and extension (72 °C for 15 sec). One positive control for each genotype (CC, CT, and TT) and 1 appropriate negative control were included in each run. The positive controls were verified by using the PCR with the restriction fragment length polymorphism (RFLP) technique as described previously by Somekawa et al.^19^ with minor modifications. The genotypes were separated by electrophoresis on 2% agarose gel stained with ethidium bromide (Figure 1) and also confirmed by direct DNA sequence analysis. Following the PCR amplification steps, melt curves for the products were generated by heating in 0.1 °C increments at a rate of 2 seconds per each step over the temperature range from 65 °C to 95 °C. The HRM data were analyzed using the Rotor-Gene Q software package supplied with the instrument. Sequence variations were distinguished from wild-type samples by the different shapes of normalized and temperature-shifted melting curves (Figure 2). In samples which failed to give an interpretable HRM pattern (~10%), the PCR-RFLP method was used for genotyping and the results were reconfirmed by direct sequencing in 15 samples.

M, 50 bp marker ladder; Lanes 2, 3, 5, and 9, CC genotype; Lanes 6, 8, and 11, CT genotype; Lanes 1, 4, 7, 10, and 12, TT genotype.

The continuous variables are expressed as means ± standard deviations (SDs) and the categorical variables as frequencies (%). The case and control groups were compared using the independent 2-sample Student t-test (or the Mann–Whitney U-test, if required) for the continuous variables and the χ2 test (or the Fisher exact test, as appropriate) for the categorical variables. A logistic regression model was constructed to test the independent relationship between the rs1800588 variants and the presence of CAD and the SNP coded as 0, 1, or 2 based on the number of risk (T) alleles. The analyses were repeated after adjusting for diabetes mellitus, cigarette smoking, hypertension, family history of CAD, and the body mass index (BMI) as well as serum HDL-C, triglyceride, and low-density lipoprotein cholesterol (LDL-C) in an established multivariable model. Odds ratios (ORs) and 95% confidence intervals (CIs) were calculated in all the patients and in the males and females separately. After the exclusion of 503 patients with normal coronary arteries (Gensini score = 0), a multiple linear regression model was employed to search for an independent association between the -514 C/T polymorphism genotypes and alleles and the severity of CAD as calculated by the Gensini score. As the Gensini score was not normally distributed, logarithmic (log) transformed values were used for multivariate regression analysis. The dependent variable was the natural log-transformed Gensini score, and multivariate adjustments were made for the aforementioned potential covariates. A p value equal to or less than 0.05 was considered statistically significant. All the statistical analyses were done using PASW Statistics for Windows, version 18.0. (Chicago: SPSS Inc.). 

The LIPC rs1800588 nucleotide changes resulted in different curve patterns using high-resolution melting analysis.

LIPC, hepatic lipase; CC, Wild-type genotype; CT, Heterozygous genotype; TT, Mutant homozygous genotype

## Results

One hundred forty-six patients were excluded due to missing data or genotyping failure (89 cases and 57 controls). Therefore, a total of 974 individuals were entered in the analysis (87.0%), comprising 471 cases and 503 controls. The mean age of the study participants was 45.5 ± 6.0 years and 46.1% were men. The clinical and demographic characteristics of the study sample are summarized in Table 1. The overall prevalence of cardiovascular risk factors including, hypertension, diabetes mellitus, cigarette smoking, dyslipidemia, and family history of CAD was high. The prevalence of family history of CAD, dyslipidemia, and cigarette smoking as well as diabetes and hypertension was significantly high in the CAD group compared to the controls, while there was no statistically significant difference in age, sex, and the BMI between the 2 groups. When compared to the control group, the CAD patients had high mean values of triglyceride and HDL-C, but the mean values of total cholesterol and low-density lipoprotein cholesterol (LDL-C) were similar between the 2 groups. The allele and genotype frequencies for the -514 C/T (rs1800588) polymorphism in the non-CAD and CAD patients, separated by gender, are demonstrated in Table 2. There was no statistically significant difference in the distribution of the -514 C/T genotypes between the 2 groups in the whole population. After stratification by sex, the examined polymorphism was found to be associated with the presence of CAD in the women (p value = 0.029) but not in the men (p value = 0.651) in the univariate analysis. 

**Figure 3:**
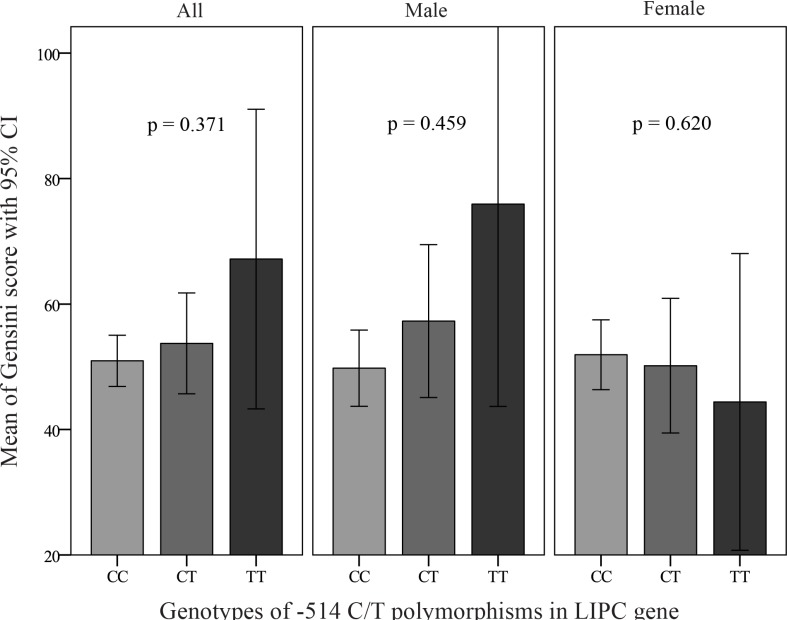
Effects of the different genotypes of -514C/T polymorphisms in the hepatic lipase (LIPC) gene on the Gensini score in the coronary artery disease (CAD) group (n = 471) and in the men and women subgroups. P values are adjusted for diabetes, hypertension, body mass index, family history of CAD, and smoking, as well as plasma levels of low-density lipoprotein (LDL) cholesterol, high-density lipoprotein (HDL) cholesterol, and triglyceride. CAD, Coronary artery disease

**Figure 4:**
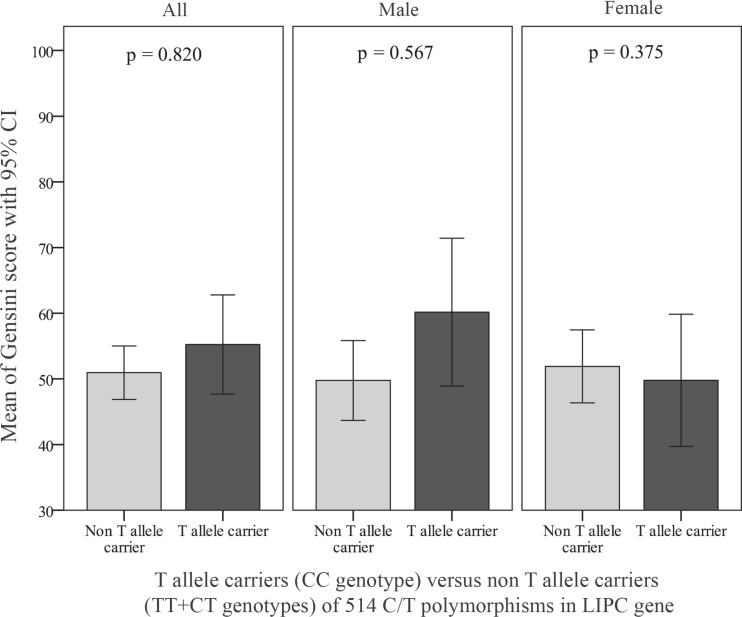
Comparison of the average Gensini score between the T allele carriers ([CC] genotype) and the non T allele carriers ([TT]+[CT] genotypes) of 514C/T polymorphisms in the LIPC gene among all the CAD patients and in the men and women subgroups. P values are adjusted for diabetes, hypertension, body mass index, family history of coronary artery disease, and smoking, as well as plasma levels of low-density lipoprotein (LDL) cholesterol, high-density lipoprotein (HDL) cholesterol, and triglyceride.

**Figure 5:**
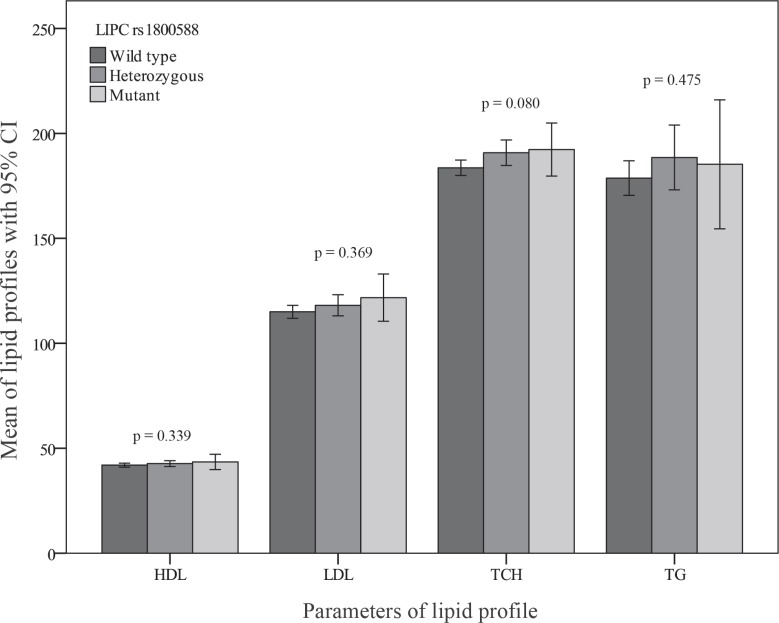
Mean lipid concentrations according to the -514 C/T genotype of the hepatic lipase (LIPC) gene.

HDL, High-density lipoprotein cholesterol; LDL, Low-density lipoprotein cholesterol; TCH, Total cholesterol; TG, Triglyceride

Table 3 shows that after the application of a multiple logistic regression model adjusting for diabetes mellitus, cigarette smoking, hypertension, family history of CAD, and the BMI as well as serum HDL-C, triglyceride, and LDL-C, the examined polymorphism was not found to be associated with the presence of CAD in the total population or in the women and men separately. Moreover, after the exclusion of 503 patients with normal coronary arteries (Gensini score = 0), the association between the -514 C/T polymorphism and the Gensini score, which effectively reflects the severity of coronary stenosis, was assessed among a total number of 471 patients with CAD. By multiple linear regression with the natural log-transformed Gensini score as a dependent variable, there was no significant association between the examined polymorphism and the severity of CAD in the total population and in the subgroups of men or women (Figures 3 and 4). In addition, Figure 5 shows that no associations were detected between the LIPC rs1800588 (C > T) genotypes and the lipid profile including total cholesterol, HDL-C, LDL-C, and triglyceride levels. Even after the analysis of the data separately for the men or women as well as for the CAD or non-CAD subgroups, this SNP in the LIPC gene was not associated with the lipid profile (data not shown).

**Table 2:** Genotype and allele frequencies of the -514 C/T polymorphism in the hepatic lipase (LIPC) gene and its relationship with CAD in the whole study population and subgroups separated by gender*

	Non-CAD Patients			CAD Patients			P value (Non-CAD vs. CAD)		
	All (n=503)	Male (n=225)	Female (n=278)	All (n=471)	Male (n=224)	Female (n=247)	All	Male	Female
-514 C/T							0.931	0.651	0.029
CC	333 (66.2)	146 (64.9)	187 (67.3)	311 (66.0)	140 (62.6)	171 (69.2)			
CT	142 (28.2)	70 (31.1)	72 (25.9)	142 (30.1)	71 (31.7)	71 (28.7)			
TT	28 (5.6)	9 (4.0)	19 (6.8)	18 (3.8)	13 (5.8)	5 (2.0)			
CT + TT	170 (33.8)	79 (35.1)	91 (32.7)	160 (34.0)	84 (37.5)	76 (30.8)	0.955	0.559	0.630
Alleles									
C	808 (80.3)	362 (80.4)	446 (80.2)	764 (81.1)	351 (78.3)	413 (83.6)			
T	198 (19.7)	88 (19.6)	110 (19.8)	178 (18.9)	97 (21.7)	81 (16.4)			

CAD, Coronary artery disease

* Data are presented as n (%).

**Table 3:** Multiple logistic regression model for the determination of the independent effect of-514C/T genotypes on premature coronary artery disease in the whole study population and in men and women separately

-514 C/T genotypes	Unadjusted		Adjusted*	
	Odds ratio (95% CI)	P value	Odds ratio (95% CI)	P value
All participants		0.395		0.565
CC	1.0 (Ref.)	-	1.0 (Ref.)	-
CT	1.071 (0.810-1.416)	0.631	1.075 (0.786-1.470)	0.650
TT	0.688 (0.373-1.269)	0.232	0.730 (0.365-1.460)	0.374
CT+TT	1.008 (0.773-1.314)	0.955	1.022 (0.758-1.378)	0.887
Allele T vs. C	0.951 (0.759-1.191)	0.661	0.969 (0.752-1.248)	0.807
Male sex		0.654		0.690
CC	1.0 (Ref.)	-	1.0 (Ref.)	-
CT	1.658 (0.707-1.583)	0.785	1.087 (0.699-1.689)	0.711
TT	1.506 (0.624-3.635)	0.362	1.513 (0.565-4.049)	0.410
CT+TT	1.109 (0.755-1.629)	0.599	1.133 (0.743-1.729)	0.561
Allele T vs. C	1.137 (0.822-1.571)	0.438	1.151 (0.807-1.640)	0.437
Female sex		0.043		0.156
CC	1.0 (Ref.)	-	1.0 (Ref.)	-
CT	1.078 (0.732-1.589)	0.703	1.040 (0.662-1.635)	0.865
TT	0.288 (0.105-0.788)	0.015	0.336 (0.108-1.044)	0.059
CT+TT	0.795(0.579-1.091)	0.156	0.809 (0.560-1.169)	0.258
Allele T vs. C	0.913 (0.632-1.320)	0.630	0.905 (0.588-1.392)	0.650

CI, Confidence interval; Ref., Reference category; CAD, Coronary artery disease; BMI, Body mass index; HDL-C, High-density lipoprotein cholesterol; LDL-C, Low-density lipoprotein cholesterol

* Adjusted for diabetes mellitus, cigarette smoking, hypertension, family history of CAD, and BMI as well as serum HDL-C, triglyceride, and LDL-C

## Discussion

The proximal promoter of the LIPC gene contains 4 polymorphic sites (-250 G/A, -514 C/T, -710 T/C, and -763 A/G) in complete linkage disequilibrium.^11^ The most frequently studied SNP is the -514 C/T, which has been linked to a low LH activity and high concentrations of HDL-C.^12^ The lower HL activity and higher levels of HDL-C, associated with the -514 C/T variant, may at least theoretically result in a lower risk of CAD. In the present study, we evaluated the effects of the -514 C/T (rs1800588) polymorphism on CAD presence in a relatively large Iranian sample of patients with premature CAD. We found no association between the examined SNP and the presence and severity of premature CAD.

Previous studies on the association between polymorphisms in the LIPC gene and atherosclerotic cardiovascular disease have produced discordant results.^20^ Several studies have demonstrated no association,^21^^-^^26^ whereas others have shown higher risks of CAD for the carriers of the -514T allele.^12^^, ^^27^^-^^32^ In line with the result of the present study, the findings of a recent meta-analysis of 11,906 cases and 13,273 controls from 18 published case–control studies suggested that the G-250A and C-514T polymorphisms of the LIPC gene were not associated with CAD susceptibility.^33^ Similarly, the Copenhagen City Heart Study, a cohort study of the Danish general population, in nearly 9,000 participants showed no association between 6 LIPC genetic variants and elevated levels of HDL-C and the risk of ischemic cardiovascular disease.^34^ In a group of young CAD patients (CAD in men and women aged ≤ 50 y), Ji et al.^28^ suggested that being a carrier of the T allele was associated with increased risks of premature CAD in men but not in women. On the other hand, in a large population-based Danish study, homozygosity for 3 common SNPs located in the LIPC promoter region (−216A, −480T, and −729G) was related to a higher risk of ischemic heart disease despite higher HDL-C levels.^29^ Likewise, in a most recent study involving 980 Mexican patients with fatty liver and 488 controls, an association was also detected between the -514T allele and subclinical atherosclerosis measured by coronary artery calcification.^10^ Among Iranian samples, while one study 15 found no difference in the frequency of the -514T allele between CAD patients and those with normal coronary arteries in patients with general CAD, another study on patients with type II diabetes^35^ showed a significant association between the CT genotype of the - 514 C/T polymorphism and CAD risk. 

The association between the LIPC gene polymorphisms and the lipid profile is also complex. We found no associations between the LIPC rs1800588 (C > T) genotypes and the lipid profile in our studied patients. Similarly, Todur et al.^36^ did not find such associations in the CAD group; nevertheless, in their healthy controls, HDL-C and HDL3-C subfraction showed an elevation in T allele carriers. Several previous studies have examined the association between the LIPC C-514T polymorphism and the plasma lipid profile with different results according to gender and ethnicity. Most of them have confirmed the association with regard to HDL-C while as to triglyceride, total cholesterol, and LDL-C, most studies have presented nonsignificant results^.^^37^

A significant part of the discrepancies in the results of different studies may be explained by the dissimilarities in the definition of phenotype, inadequate sample sizes, and differences in the genetic background and ethnicity of participants. A precise definition of the phenotype, which is an important issue in designing genetic studies, is especially essential in studies on CAD association because individuals with significant CAD who are clinically silent may be misclassified as controls, resulting in a higher likelihood of null results. To avoid this bias, we defined phenotype on the basis of objective angiographic documentation of coronary artery status. Moreover, Wang et al.^33^ concluded that the impact of a single genetic factor on CAD risk could be more prominent when there were such other common genetic or environmental cardiovascular risk factors as obesity, smoking, hypercholesterolemia, and diabetes.^38^ It should also be noted that contrary to expectations, the LIPC -514T allele has not been previously shown to be inversely associated with CAD. Actually, the elevated HDL-C in minor allele carriers also has been linked with the highest CAD risk.^13^^, ^^29^ This could be because of a modulation in the HDL particles induced by HL activity.^39^

We observed no association between the rs1800588 (-514C > T) SNP in the LIPC gene and the Gensini score as a semiquantitative index of the severity of coronary atherosclerosis. This is in accordance with a previous study by Chen et al.,^40^ who reported that the -514C > T SNP was not associated with the minimal lumen diameter of coronary atherosclerotic lesions or the mean number of coronary artery occlusions at the baseline. However, it has been shown that this polymorphism in the HL promoter is associated with a higher extent of CAD.^13^^, ^^30^ Hokanson et al.^41^ suggested that in patients with type 1 diabetes, the LIPC -480C > T polymorphism was associated with the extent of coronary calcification in a dosage-dependent manner. 

The strength of our study is that we included a relatively large cohort of Iranian patients with premature CAD, well documented by coronary angiography. This afforded us the unique opportunity to assess genetic effects. However, this study has several limitations. First, as a result of our case–control design, a selection bias may have been introduced. Second, the control group may not represent a healthy general population because the controls were selected from individuals with suspicious CAD on clinical assessment who subsequently underwent coronary angiography. Finally, our study, albeit relatively large, was not sufficiently powered to detect small to modest effect sizes.

## Conclusion

We did not find any significant relation between the LIPC -514 C/T polymorphism and the risk of premature CAD among a relatively large number of Iranian patients undergoing coronary angiography. Given the limitations of simple association studies, further larger studies are required to assess such associations in different ethnic populations. 

## Notes:

***This paper should be cited as:*** Goodarzynejad H, Boroumand M, Behmanesh M, Ziaee S, Jalali A, Pourgholi L. Association between the Hepatic Lipase Promoter Region Polymorphism (-514 C/T) and the Presence and Severity of Premature Coronary Artery Disease. J Teh Univ Heart Ctr 2017;12(3):119-127.
